# Belladonna vs. Opium

**Published:** 1874-12-01

**Authors:** O. T. Maxson

**Affiliations:** Waukegan


### BELLADONNA vs. OPIUM.

EDITORS EXAMINER: The following case may be of interest as additional testimony of the efficacy of belladonna in opium poisoning.

Mr. P——, aged sixty-four, of delicate and feeble health, designing to relieve himself of the cares of this life, wrote his own obituary, gave directions for his secular affairs, bade good-by to his friends, swallowed eight grains of sulph. morphia, took to his bed, and considered himself ticketed out of trouble. He took morphia between the hours of 12 M. and 2 P. M.; was not discovered until 6 P. M. to be unwell, being alone until that time, and then considered to be only in a heavy sleep, and was allowed to remain until 9 P. M., when I was called. I saw him at 10, eight to ten hours after taking the morphia. I found him in a deep sleep; stertorous breathing; respirations eight to ten per minute; pulse 60, full and hard; almost total insensibility to external impressions. External irritants, violent agitation, cold douche, moving him about the room, supported by two strong men, he making no effort to support himself, were resorted to without any change from the deep coma. Electricity and coffee were tried; very little coffee could be swallowed. Receiving no hope from the above I laid him upon the bed and gave him a large teaspoonful of tr. of belladonna, at 11 P. M. At the end of thirty minutes, finding no change, repeated belladonna. In twenty minutes, decided enlargement of the pupils, breathing better; hands and feet that had been cold and clammy, sweating, gave evidence of returning warmth. At 12 gave third dose of belladonna. 12.30, pupils twice ordinary size; pulse 80, much less in force and volume; hands and feet dry, breathing better; unable to make him give evidence of consciousness to external irritation. 2 P. M., gave one-half teaspoonful belladonna. At 3 P. M. pulse 100, moderate in force; breathing much better; ordered carb. ammonia, 5 grains in a little sweetened water, to be repeated every hour with beef tea, and instructions to keep him warm; left with orders to call me if any change before morning. At 7 A. M. found him breathing easy; would withdraw his hand from electro-magnetic current; drank cup of coffee; continued stimulants. At 12 M. would make efforts to answer questions; hands and feet warm; considerable prostration. At 2 desired to make water, and stood upon his feet to make the effort; did not pass any urine. Left him with instructions to continue ammonia and watch for any lack of circulation, to call me on any new development. Reported to have passed a quiet night; next morning insisted upon going to the table, and walked out and ate with the family, still showing great prostration. Having my time fully taken up, and assured I should be notified if not doing well, also a member of the family having visited me and reported favorably, I did not again visit him. At this time his medicine gave out. They, thinking he did not need more, did not report to me. During the following afternoon he showed great prostration; at 6 P. M. hands and feet cold, pulse reported very low. During all this time no medication was given. At 8 P. M. he died. I think the belladonna is entitled to the credit of having passed him over the stupor of the morphia, and that had suitable stimulants been given during the stage of nervous prostration, he would have recovered from the injury of the opium poison.

O. T. MAXSON, M. D.

Waukegan, Nov. 15th, 1874.

---

PERIOSTEAL FLAPS IN AMPUTATIONS.—Tizzoni believes that the results of numerous operations on animals and occasional examinations of the stumps of patients, justify him in making the following statements as to the advantage of periosteal flaps:

1. The operation is easily performed. At the present day, when pain and blood is spared the patient, it is a matter of small consequence if a little more time is consumed than usual. 2. The periosteal flaps retract considerably, but equally and without wrinkles. 3. These flaps will remain spread over the cut surface of the bone. 4. In twenty-four hours there will be a firm adhesion between the medulla and the flaps. Later, they consolidate with the bone. This early adhesion is of the greatest practical significance. It protects the medulla from the influence of the pus that may possibly form about the end of the bone.—*Riv. Clin. d. Bologna*, *Allg. Med. Central Ztg.*, 74, 1874.

